# A Novel Method to Maintain the Sample Position and Pressure in Differentially Pumped Systems Below the Resolution Limit of Optical Microscopy Techniques

**DOI:** 10.1177/0003702820942798

**Published:** 2020-10-06

**Authors:** Christopher M. Goodwin, John D. Alexander, Matthew Weston, David Degerman, Mikhail Shipilin, Patrick Loemker, Peter Amann

**Affiliations:** 1 Department of Physics, Stockholm University, AlbaNova University Center, Stockholm, Sweden; 2 Photon Science, Deutsches Elektronen-Synchrotron DESY, Hamburg, Germany

**Keywords:** Ambient pressure XPS, differentially pumped systems, constant distance, feedback loop, distance control, pressure control, proportional–integral–derivative loop, PID loop, in situ

## Abstract

We present a new method to maintain constant gas pressure over a sample during in situ measurements. The example shown here is a differentially pumped high-pressure X-ray photoelectron spectroscopy system, but this technique could be applied to many in situ instruments. By using the pressure of the differential stage as a feedback source to change the sample position, a new level of consistency has been achieved. Depending on the absolute value of the sample-to-aperture distance, this technique allows one to maintain the distance within several hundred nanometers, which is below the limit of typical optical microscopy systems. We show that this method is well suited to compensate for thermal drift. Thus, X-ray photoelectron spectroscopy data can be acquired continuously while the sample is heated and maintaining constant pressure over the sample. By implementing a precise manipulator feedback system, pressure variations of less than 5% were reached while the temperature was varied by 400 ℃. The system is also shown to be highly stable under significant changes in gas flow. After changing the flow by a factor of two, the pressure returned to the set value within 60 s.

## Introduction

The development of efficient catalysts for chemical production is essential to develop a carbon-neutral economy.^1^ To understand the reactions that occur on potential catalysts, in situ spectroscopic observation of the catalyst is required. Although many forms of spectroscopy can shed light on how these catalysts work, few provide quantitative atomic information on the surface, which is necessary to understand the mechanism of the catalyst. Without direct observation of the atomic mechanism, the development of a new catalyst becomes much more difficult. Towards this end, the pressure gap, i.e., the separation of typical atomic-scale spectroscopy and industrially relevant reaction conditions, has to be bridged.^2–4^

The recent developments of in situ atomic-scale spectroscopy are essential to understand the reaction mechanisms of catalytic,^5^ environmental,^6^ electrochemical,^7^ and many other reaction types. The traditional problems caused by the pressure gap, such as unknown reaction intermediates, are being solved.^8^ Meanwhile, old and new issues have arisen from the development of new methods to gather in situ spectra such as: thermal stability,^9^ accurate pressure measurements,^10^ and cleanliness.^11^

Many groups have built state-of-the-art in situ X-ray photoelectron spectroscopy (XPS),^12–15^ scanning tunneling microscopy (STM),^16^ transmission electron microscopy (TEM),^17^ and even secondary ion mass spectrometry^18^ systems to study surfaces under reaction conditions. In all these instruments, the relative position of the sample to the detector has a significant bearing on the measurements. Further complications develop with sample heating and the resulting thermal expansion, which is a standard experimental variable. Charged particles such as electrons and ions have a high scattering cross-section and, consequently, a low inelastic mean free path (IMFP) in gases. The IMFP is dependent on the kinetic energy of the charged particle and also of the gas type that the particle is penetrating through. For XPS conditions of 3 keV photon energy and a gas pressure of 100 mbar N_2_, the IMFP is approximately 20 µm.^19^ A short IMFP necessitates the use of differentially pumped analyzers, where the sample is placed in an environment of elevated pressure, and an aperture that leads to a detector is brought close to the sample surface. The aperture restricts the number of gas molecules that diffuse towards the detector and allows the implementation of differential pumping behind the aperture.^20,21^

While the method presented in this article is, in general, applicable to differentially pumped systems, we demonstrate it using a new XPS setup following the concept of a virtual cell.^13^ This approach creates a local high-pressure region where pressure is strongly dependent on the sample-to-aperture distance.

One of the most significant problems with using a differentially pumped analyzer is the effect that the sample-to-aperture separation has on pressure. Typically, this has been treated as a problem that must be overcome to be able to carry out in situ experiments. Herein, a method is established to make use of the effect that the sample distance has on the pressures of differentially pumped stages. Furthermore, it is outlined how one can create a feedback loop to maintain the sample separation, thereby converting one of the most substantial problems in in situ research into an advantage.

## Background

One of the most commonly implemented techniques available to perform in situ measurements that require charge detection is to use a differentially pumped analyzer. In situ measurements typically consist of multiple differentially pumped stages to decrease the pressure from the sample to the detector. Often this is combined with a sophisticated lens system for efficient transfer of charged particles.^20,21^ The aperture diameter is chosen as a tradeoff between high transmission (large aperture) and high pressure (small aperture). To achieve higher pressures, the open area of aperture is decreased, which in turn also allows for shorter sample-to-aperture distance.^10,22,23^ The general procedure is to keep the sample at a distance of between one to three times the aperture diameter away from the aperture; this guarantees that the pressure is still within 90% of the surrounding chamber pressure.^24^ Reasonable distances for lab-based systems are typically in the range of 500–800 µm,^14,25–27^ while for synchrotron applications this distance can be reduced to 150–300 µm^28^ and for some specific cases, including the system used herein, down to 30 µm.^13,29^ At these distances, a small change in distance of 1 µm can have a substantial effect on the pressure over the sample, the pressure in the differentially pumped section, and the signal from the sample. To date, few systems have been able to achieve pressures over 50 mbar. However, the trend in experimental development clearly shows the intention to push this limit. Therefore, to achieve higher pressure over the sample, the distance between the sample and aperture needs to be decreased further; therefore, distance variations would have a more significant effect.

Optical systems are naturally utilized for tracking the sample position inside experimental setups. Small distances, however, represent a challenge for the positioning as it comes close to the wavelength of optical light and therefore is close to the limit of what can be achieved in terms of resolution in optical microscopy. Furthermore, microscopes rely heavily on how well the region is illuminated, which is very challenging in a typical practical apparatus. Alternative distance measurement systems such as capacitance gauges cannot be implemented directly on the sample surface but are only possible on reference surfaces. Unfortunately, reference surfaces are generally not subject to the same thermal expansion as the sample, limiting the level of control provided.

Controlling the sample-to-aperture distance at a value of several 10–40 µm and maintaining it within 1 µm is experimentally challenging. Thermal expansion and vibrations need to be considered in the design. Long transfer arms and systems without a dedicated analysis stage can have significant vibrations; the vibrational stability is a considerable concern for STM and TEM instruments. Still, it is also a potential problem as higher pressures are sought with in situ systems. A much more complex concern is the effects of thermal drift.^24^ The vast majority of the experiments that are of interest for in situ research are reliant on the effects of temperature on the reactions. As a result, the most common failure of an in situ experiment is due to the impact of thermal expansion and contraction of the sample, sample holder, front cone, and manipulator. Although some systems can employ thermal loops that can avoid the problem of thermal expansion, the typical system is simply too complex and intricate to be able to mount the sample on a highly complex stage or directly to the aperture housing. The complexity is primarily due to the use of bulky energy sources that cannot be modified, such as synchrotron X-ray beams. While some precautionary measures can be taken to reduce thermal expansion, even a few microns change in the sample-to-aperture distance would have a substantial effect on the pressure. The precautions that can be made to minimize thermal expansion include choosing the mounting points so that when heating parts expand away from the aperture and to limit the elements that will get hot by thermally isolating ceramics. With these measures, the system herein has had a thermal expansion of about 9 µm per 100℃.

The commonly used approach to maintain sample distance for in situ research is to use the detector response as the feedback for sample positioning. For ambient pressure XPS (AP-XPS), this would mean monitoring a photoelectron peak intensity. This method comes with many problems; the first is that it requires the experiment to be halted to evaluate the sample position. Stopping acquisition greatly extends the total experimental time; due to this, the sample position is often neglected until a problem is identified. Although this approach can be useful, it does require a problem to be noticed by careful observation before it can be addressed. Another major problem with using the detector to maintain separation is that by choosing the wrong spectral component, a problem can go unseen. This second case is when the chemical nature of the sample is changed due to the reaction; the most common example is when an oxide or carbon film grows or is depleted from the surface. If the user is not careful to observe multiple spectral components, it is possible for the sample to move from the ideal position without a spectral change in the observed species or a worse case where the sample is moved due to an actual chemical change that was not expected. The third problem with using the detector in the feedback loop is that there is no way to guarantee that the sample’s position is maintained within one spectrum, which is especially problematic for long-term acquisition.^13^ Thermal expansion takes place over various time domains; the heater, sample, and sample holder are subject to short-time domains changes of minutes, but the manipulator and transfer rod change the sample position over hours.

The problems expressed above point to a need for a method to maintain the sample-to-aperture separation. For all differentially pumped systems, the sample-to-aperture distance is monotonically related to the pressure in the differential pumping stage.^13,30^ Therefore, maintaining a constant pressure in the first differential pumping stage also maintains a constant sample-to-aperture distance. A pressure gauge in this stage is an ideal source of information for developing a position feedback loop, to maintain a constant separation due to the pressure depending on the sample-to-aperture distance yet being measured independently. By using a feedback input that is typically independent from the experimental variables of interest, a feedback loop can be implemented to increase the reliability and accuracy of the sample’s position significantly.

**Figure 1.**
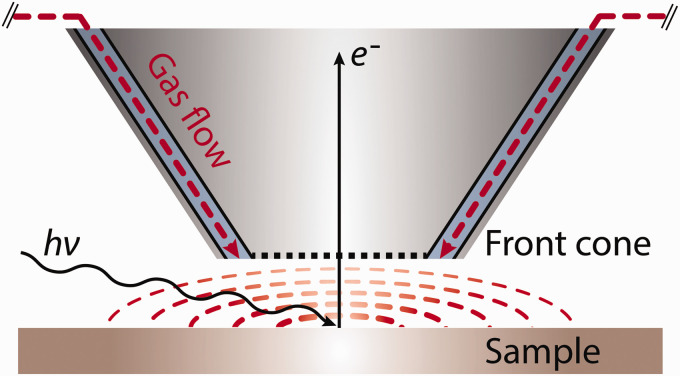
A mockup of the sample and aperture, indicating that the gas is approaching the sample from within the analyzer cone. Typical separation under reaction conditions is 30 µm. For more details, see Amann et al.^13^

Figure 1 shows a mockup of the sample and aperture, indicating that the gas is approaching the sample from within the analyzer cone. Typical separation under reaction conditions is 30 µm; for more details, see Amman et al.^13^

## Variables Affecting the Sample Pressure

Figure 1 shows the sample and front cone geometry (not to scale) and how the electron signal is generated and gathered, with a typical spacing between them of 30 µm. The aperture is part of the bottom of the front cone; this is where the electrons enter the analyzer. In this design, a high-pressure zone is created on the sample surface using the gas flow from the inlet nozzles (see Amann et al.^13^ for more details). Figure 1 shows the input gas flow in dashed red lines and the high-pressure zone displayed by the red hatch circles. Gas flows inwards from the outer ring towards the center, then through the aperture into the differentially pumped lens and to the back of the chamber. From this, it is clear that the pressure above the sample is dependent upon the distance to the aperture, gas flow, and gas composition, see Amann et al.^13^ for a more detailed discussion. For the measurements discussed herein, a sample of polished polycrystalline titanium was used. The sample was mounted on a resistive heater in a custom-made holder. A thermocouple was installed under the sample, between the heater and the sample, and a second thermocouple was spot-welded to the top of the sample where it would not make contact with the front cone at small separations.

**Figure 2.**
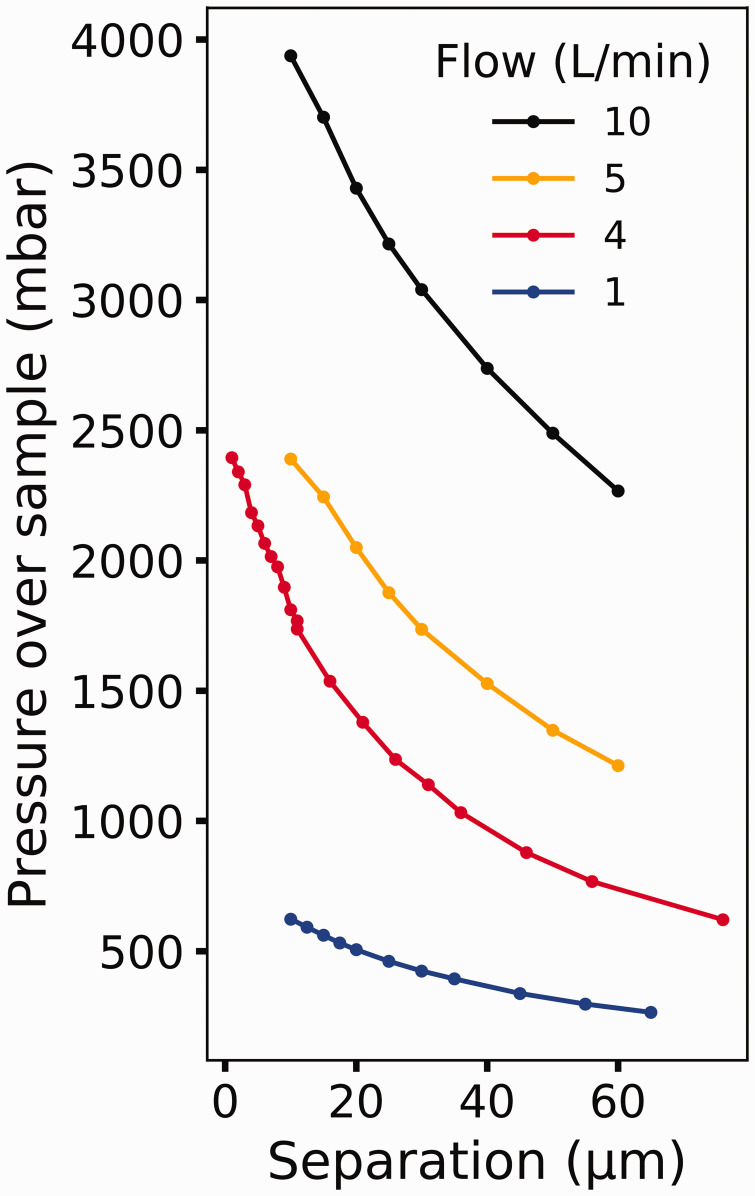
The effect that sample-to-aperture separation and flow has on the pressure that the sample experiences. The indicated flows of 1, 5, and 10 L/min have been conducted using CO_2_ gas, and the 4 L/min flow was done using oxygen gas.

Figure 2 shows how the pressure changes as a function of the separation between the sample and the first aperture. The *y*-axis is based on the calibration of the first stage pressure when the chamber is pressurized with the sample fully retracted, as shown previously.^13^ From Fig. 2, it is shown that the pressure is monotonically dependent on the sample-to-aperture distance showing an exponential-like behavior as distance changes, making it an ideal input for a feedback loop.^31^ Due to the nature of the relationship between separation and pressure, the feedback loop is based on the log of the pressure, as is typical for exponentially dependent proportional–integral–derivative (PID) systems. The maximum achievable accuracy in pressure is thereby determined by the smallest motion that can be made. Movements as little as 100 nm are readily achievable; with a separation of 10 µm and a flow of 4 L/min (where L stands for liters at atmospheric pressure) a pressure change of 5 mbar can be made.

## Feedback Scheme

Since pressure is strongly related to separation, one can use the direct input from the pressure gauge to maintain the separation. Furthermore, by using a pressure gauge that measures pressure directly, one can avoid gas selectivity and have greater confidence in the pressure for mixed gasses as shown by Schnadt et al.^15^ To this end, a ceramic capacitance manometer (Pfeiffer Vacuum Technology AG, Germany, part number: CMR 375) was used, which has a range of 10^–5^ to 0.1 mbar and a resolution of 0.003% of the full scale. The accuracy of the capacitance gauge is maintained by mounting the component horizontally and to keep a large physical separation between the gauge and any heat source. To position the sample, a highly accurate movement system is needed; to this end, a piezo motor hexapod stage was used (Symétrie Nanopos SARL, France, custom-designed).

The pressure change in the differential stage has a time delay between changes in distance and changes in sample pressure creating a significant dead time; this is due to the complexity of how the gas flow through the aperture and pressure over the sample interact at such high pressures. This delay causes the derivative gain to make the feedback loop less stable; therefore, the feedback loop does not use a derivative gain. Finally, as mentioned above, the log of the pressure is used as the feedback variable due to the exponential, like the relationship between separation and pressure.

## PID Stability

### Stability Under Changes in Temperature

**Figure 3.**
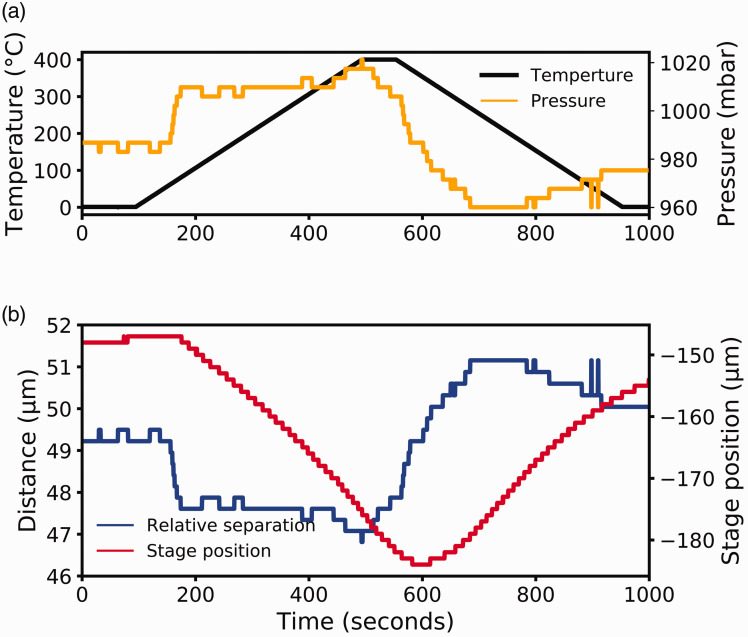
The stability of the PID loop as temperature changes. (a) The temperature and pressure profile of the sample. (b) The motion of the sample.

To demonstrate the stability of the feedback loop, the temperature of the sample was varied. Figure 3 shows how the feedback loop performs while the temperature changes. For these measurements, the temperature was ramped from room temperature to 400 ℃ and back to room temperature at a ramp rate of 1 ℃ per second using a resistive heater, with a CO_2_ gas flow of 4.0 L/min. The nominal gap distance was chosen to be 50 µm. The PID loop explained above was enabled to maintain the sample pressure, counteracting the thermal expansion. Figure 3a shows the sample temperature (black line, plotted on the left *y*-axis) and the pressure over the sample (orange line, plotted on the right *y*-axis). Figure 3b shows the relative separation between the aperture and sample (blue line, plotted against the left axis) with the relative stage position (red line, plotted on the right axis). The relative separation is based on the pressure distance curves, like those shown in Fig. 2. It is shown that the stage moves over 35 µm, yet the pressure does not vary more than 10%, and the standard deviation of the relative separation is less than 5%. From this, it is seen that when the temperature is changed by over 370 ℃, the sample pressure recovers within three minutes with a variation of less than 5%. As the feedback loop includes an integral term, the variation will eventually reduce to a value close to zero for extended periods. Thermal motion, where the sample expands or contracts from heating or cooling, is the most realistic scenario since, in practice, the pressure is set and easy to maintain using the feedback loop. Still, the temperature change can affect the pressure, as discussed above.

### Stability Under Changes in Flow

**Figure 4.**
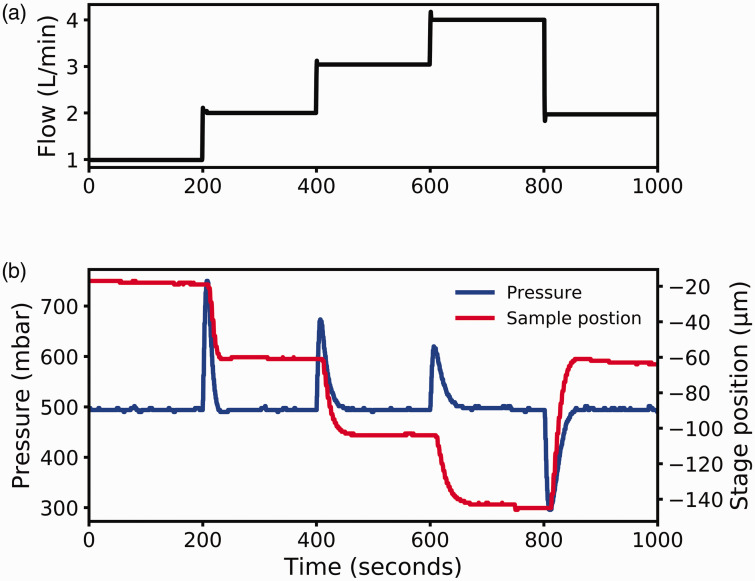
Stability of the PID loop as flow changes. (a) The flow profile. (b) The pressure over the sample and motion of the sample.

To determine the stability of the PID loop in extreme conditions, the flow of gas over the sample was varied. Figure 4 shows how quickly the feedback loop alters with changes in flow. Figure 4a shows the flow profile used in black. The sample was heated to 400 ℃ and with a nominal pressure of 500 mbar. Figure 4b shows the response of the PID loop where the pressure over the sample is shown in blue plotted against the left axis and the red line showing the relative stage position plotted on the right axis. Figure 4 shows that the PID can respond to extensive changes in flow, much larger than typically needed, and still return to a stable state within 60 s.

## Effectivity

**Figure 5.**
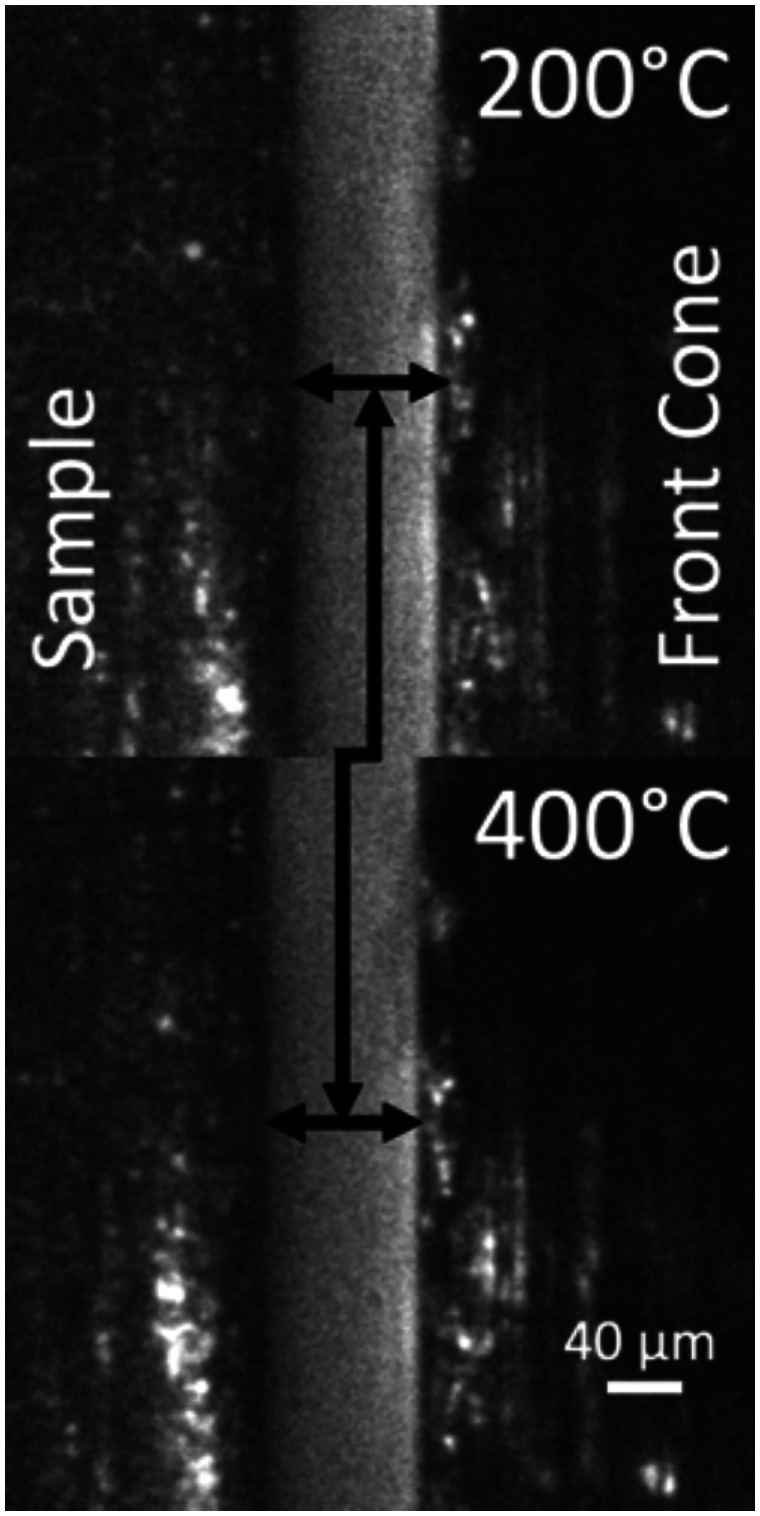
Effect of heating on sample motion and the negation of the thermal movement by the PID. The sample is shown on the left and the front cone on the right.

As shown above, the distance between the sample and the aperture determines the pressure above the sample. The relative separation is influenced by experimental variables that are probed during a reaction. The most common variable that can affect the separation is the temperature which changes when the sample is heated or cooled. The source or sink of heat at the sample will also affect the entire manipulator which will subsequently also thermally expand or contract. The front cone can also be heated from the heat radiating off of the sample, expanding the front cone towards the sample. Figure 5 shows the sample on the left and the aperture on the right, both expanding as the sample was heated from 200 ℃ to 400 ℃ while under a flow of 4.0 L/min and a pressure of 500 mbar. The total motion was negated by the PID loop, a relative movement of 20 µm with a nominal separation of 75 µm.

The wide range of the stability of the feedback allows for fast and accurate isobars, where the temperature is swept and the pressure is held constant. Figure 3 shows that the feedback loop is stable over swift temperature changes; typical experiments require temperature changes of at most a degree a minute. The stability serves not only to maintain the pressure over the sample over a variety of standard situations such as changing temperature but also allows for previously unfeasible experiments. One such example would be to move the sample parallel to the aperture while maintaining the same reaction environment. If the sample has any non-uniformities or is misaligned, then moving the sample would change the distance, and therefore the pressure, over the sample, but the PID loop would account for this. Another possibility is to change the gas composition, usually not possible since mass flow controllers only account for mass passed by; therefore, if the gas mixture changes, the total pressure is typically changed. By using the PID loop, the total pressure is maintained as described before, resulting in the pressure over the sample being maintained within the viscosity deviation of the different gas compositions. Therefore, the PID loop can be used to change the chemical composition without changing the pressure. A final example of an experiment that is now possible would be to measure a sample with a dynamic surface that changes on the micron scale, such as liquids in motion.

While flow is less commonly used as an experimental variable, it is still of value to see how the feedback loop performs. Changing the flow can be used to evaluate the mass transfer limit, gas cleanness, and the effects of small thermal changes. The changes in gas flow also show that if there were to be a sudden change in the gas flow, the feedback loop could still maintain the pressure. In practice, the most significant concern in this feedback system is the flow decreasing due to the gas supply running out. Without a gas flow, the sample would be brought in contact with the front cone in an attempt to maintain the set pressure, to prevent this, the feedback loop has a limit on the maximum possible sample displacement.

## Conclusion

Herein, a method to increase the consistency of in situ measurements such as AP-XPS has been shown. This new level of stability was accomplished by using pressure measurements in the first differentially pumped stage as feedback for the sample position. The first differentially pumped stage is ideal for the feedback input to maintain sample-to-aperture separation since it is exponentially dependent on the separation of the sample from the analyzer.

The main feature that was shown was the ability to change the experimental conditions without changing the pressure the sample experienced. Thermal stability is a significant advance since one of the main complications of in situ experiments is to maintain sample conditions. Furthermore, it has been shown that the sample can be held under the same pressure to within 5% of the initial value, while the temperature changes from 20 to 400 ℃ without user input. The PID feedback was also able to accommodate substantial changes in flow; the flow was changed by a factor of two and the PID system recovered the set pressure within 60 s. The increased stability in pressure also allows for increased stability in the differential pumping sections where a residual gas analyzer is placed. As a consequence, the signal in the residual gas analyzer is stabilized.

In practice, the feedback loop needs only to be able to maintain pressure over long-time domains and small temperature changes. Pressure stability is easily within the capabilities of the system shown herein and can significantly reduce the complexity of in situ measurements. Furthermore, all systems that have the sample pressure determined by the sample to aperture separation could have improved positioning stability by employing the feedback loop as described, making the method widely applicable.
